# Ezrin Inhibition Overcomes Acquired Resistance to Vemurafenib in BRAFV600E-Mutated Colon Cancer and Melanoma Cells In Vitro

**DOI:** 10.3390/ijms241612906

**Published:** 2023-08-17

**Authors:** Iris Car, Antje Dittmann, Olga Vasieva, Luka Bočkor, Petra Grbčić, Nikolina Piteša, Marko Klobučar, Sandra Kraljević Pavelić, Mirela Sedić

**Affiliations:** 1 Centre for Applied Bioanthropology, Institute for Anthropological Research, Ljudevita Gaja 32, 10000 Zagreb, Croatia; iris.car@inantro.hr (I.C.); luka.bockor@inantro.hr (L.B.); m.klobucar186@gmail.com (M.K.); 2 Functional Genomics Center Zurich, ETH Zurich, Winterthurerstr. 190, Y59 H38, 8057 Zurich, Switzerland; antje.dittmann@fgcz.ethz.ch; 3 INGENET Ltd., 27 Market Street, Hoylake, Wirral CH47 2BG, UK; ovasieva@ingenets.com; 4 Faculty of Medicine, Juraj Dobrila University of Pula, Zagrebačka ul. 30, 52100 Pula, Croatia; petra.grbcic@gmail.com; 5 Division of Molecular Medicine, Ruđer Bošković Institute, Bijenička Cesta 54, 10000 Zagreb, Croatia; nikolina.rincic@irb.hr; 6 Faculty of Health Studies, University of Rijeka, Viktora Cara Emina 5, 51000 Rijeka, Croatia; sandrakp@uniri.hr

**Keywords:** BRAFV600E, colon cancer, melanoma, BRAF inhibitor, vemurafenib, ezrin, actin cytoskeleton, ezrin inhibitor, NSC305787

## Abstract

Despite the advancements in targeted therapy for BRAFV600E-mutated metastatic colorectal cancer (mCRC), the development of resistance to BRAFV600E inhibition limits the response rate and durability of the treatment. Better understanding of the resistance mechanisms to BRAF inhibitors will facilitate the design of novel pharmacological strategies for BRAF-mutated mCRC. The aim of this study was to identify novel protein candidates involved in acquired resistance to BRAFV600E inhibitor vemurafenib in BRAFV600E-mutated colon cancer cells using an integrated proteomics approach. Bioinformatic analysis of obtained proteomics data indicated actin-cytoskeleton linker protein ezrin as a highly ranked protein significantly associated with vemurafenib resistance whose overexpression in the resistant cells was additionally confirmed at the gene and protein level. Ezrin inhibition by NSC305787 increased anti-proliferative and pro-apoptotic effects of vemurafenib in the resistant cells in an additive manner, which was accompanied by downregulation of CD44 expression and inhibition of AKT/c-Myc activities. We also detected an increased ezrin expression in vemurafenib-resistant melanoma cells harbouring the BRAFV600E mutation. Importantly, ezrin inhibition potentiated anti-proliferative and pro-apoptotic effects of vemurafenib in the resistant melanoma cells in a synergistic manner. Altogether, our study suggests a role of ezrin in acquired resistance to vemurafenib in colon cancer and melanoma cells carrying the BRAFV600E mutation and supports further pre-clinical and clinical studies to explore the benefits of combined BRAF inhibitors and actin-targeting drugs as a potential therapeutic approach for BRAFV600E-mutated cancers.

## 1. Introduction

BRAFV600E-mutated colorectal cancer represents approximately 8–12% of metastatic colorectal cancer (mCRC) cases and is considered the most aggressive subgroup of colorectal cancer from a clinical and biological perspective [1]. In particular, the BRAFV600E mutation is associated with a significantly worse response to chemotherapy and short progression-free survival and overall survival. Thus, a recent study has shown that the 3-year overall survival rates for BRAFV600E-mutated and BRAF wild-type metastatic colorectal cancer patients were 54% and 82.9%, respectively [2]. In spite of the demonstrated therapeutic effectiveness of selective BRAFV600E inhibitor vemurafenib in BRAFV600E-mutated malignant melanoma, metastatic colorectal cancer patients harbouring the BRAFV600E mutation did not have significant clinical benefits from vemurafenib monotherapy with response rates of only around 5% [3]. The main reason for the development of primary resistance to vemurafenib in colorectal cancer was shown to be the epidermal growth factor receptor (EGFR)-mediated feedback loop leading to reactivation of the mitogen-activated protein kinase pathway that resulted in sustained cell proliferation in the presence of BRAF inhibition [4]. Clinical limitations of vemurafenib include the short duration of response, marked skin toxicities and severe photosensitivity as well as the need for long-term daily administration and the potential for drug interactions [5].

Prompted by this finding, combinatorial treatments with EGFR and BRAF inhibitors were clinically tested in BRAFV600E-mutant mCRC [6,7,8,9,10]. The BEACON trial was the first randomised phase III trial to demonstrate significantly longer overall survival and a higher response rate for the combination treatment based on anti-EGFR antibody cetuximab and BRAFV600E inhibitor encorafenib with or without the MEK inhibitor binimetinib in comparison with standard therapy in BRAFV600E-mutated metastatic colorectal cancer patients who had disease progression after one or two previous regimens [9]. Findings from this clinical trial led to the approval of the combination of cetuximab and encorafenib by the U.S. Food and Drug Administration and the European Medicines Agency in 2020 as the new standard of care for refractory BRAFV600E mCRC.

However, in spite of improved survival outcomes with the combination of EGFR and BRAF inhibitors, not all BRAFV600E-mutant mCRC patients derive clinical benefits from this treatment strategy due to relatively rapid development of secondary resistance [10,11]. In addition, the lack of established clinical standards to identify and target resistance in BRAFV600E-mutant mCRC patients on combined anti-EGFR and BRAF treatment and an absence of established treatment options following progression, hampers clinical management of these patients [1]. Therefore, elucidating the mechanisms of resistance to targeted therapies represents an essential step forward in designing and developing novel therapeutic strategies for BRAFV600E-mutant mCRC patients.

The primary aim of the current study was to identify novel protein candidates involved in the acquired resistance to the targeted BRAFV600E inhibitor vemurafenib in BRAFV600E-mutated colon cancer cells in vitro using an integrated proteomics approach. Towards this aim, we performed complementary proteomics analyses by two-dimensional electrophoresis (2-DE) coupled with MAL-DI/TOF-TOF mass spectrometry and label-free quantitative LC–MS/MS analysis. The obtained proteomics data were combined and analysed by bioinformatics tools to identify novel protein candidates potentially associated with vemurafenib resistance in colon cancer. Bioinformatics analysis of obtained proteomics data indicated actin-cytoskeleton linker protein ezrin as a highly ranked protein significantly associated with vemurafenib resistance whose overexpression in the resistant cells was additionally confirmed at the gene and protein level. Ezrin belongs to the ezrin, radixin, moesin (ERM) protein family and has a demonstrated role as a driver of tumour progression and metastatic spread whose high expression correlates with unfavourable clinical outcome in different cancer types [12,13]. Ezrin regulates cancer-cell survival and metastatic cascade by controlling cytoskeletal remodelling and cellular signalling pathways [13]. We found that ezrin inhibition by NSC305787 increased anti-proliferative and pro-apoptotic effects of vemurafenib in the resistant cells in an additive manner, which was accompanied by downregulation of CD44 expression and inhibition of AKT/c-Myc signalling. Interestingly, we also detected increased ezrin expression in vemurafenib-resistant melanoma cells harbouring the BRAFV600E mutation. Importantly, ezrin inhibition potentiated anti-proliferative and pro-apoptotic effects of vemurafenib in the resistant melanoma cells in a synergistic manner. Altogether, our study suggests a role of ezrin in acquired resistance to vemurafenib in colon cancer and melanoma cells carrying the BRAFV600E mutation and supports further pre-clinical and clinical studies to explore the benefits of combined BRAF inhibitors and drugs targeting the ezrin-regulated actin cytoskeleton as a potential therapeutic approach for BRAFV600E-mutated cancers.

## 2. Results

### 2.1. Proteomics Analyses of Vemurafenib-Sensitive and -Resistant RKO Colon Cancer Cells Carrying the BRAFV600E Mutation

We performed global proteomics analyses of vemurafenib-sensitive (parental, RKO) and vemurafenib-resistant (RKOr) cells using two complementary proteomics approaches including two-dimensional polyacrylamide gel electrophoresis (pH 3–10NL) coupled with MALDI/TOF-TOF mass spectrometry and label-free quantitative LC–MS/MS analysis. The results from 2-DE/MALDI-TOF/TOF mass spectrometry analysis revealed 127 differentially expressed proteins (DEPs) between sensitive and resistant cells with statistical significance (*p* < 0.05), among which 59 and 68 were up- and down-regulated, respectively, in the resistant cells (Supplementary Table S1). Data from LC–MS/MS analysis disclosed 125 DEPs (*p* < 0.05, log_2_(FC) > 1), among which 78 and 47 were up- and down-regulated in the resistant cells, respectively (Supplementary Table S2).

### 2.2. Bioinformatics Analysis of Proteomics Data Identifies Ezrin and Caveolin-1 as Highly Ranked Protein Candidates Associated with Vemurafenib Resistance in BRAFV600E-Mutated RKO Colon Cancer Cells

Proteins were ranked based on differential expression data obtained by 2-DE/MALDI-TOF/TOF mass spectrometry and LC–MS/MS analysis, and proteins with log_2_ (fold change ratio) and *p*-values < 0.05 were combined and subjected to bioinformatics analyses for data integration and selection of novel protein targets associated with the development of resistance to vemurafenib in RKO colon cancer cells. This selection assumed that crucial targets can be identified based on a protein functional connectivity outreach and its centrality to the protein–protein interaction (PPI) network. We accordingly constructed two PPI networks for proteins with positive fold change ratio and all differentially expressed proteins between the resistant vs. sensitive cells.

**Figure 1:**
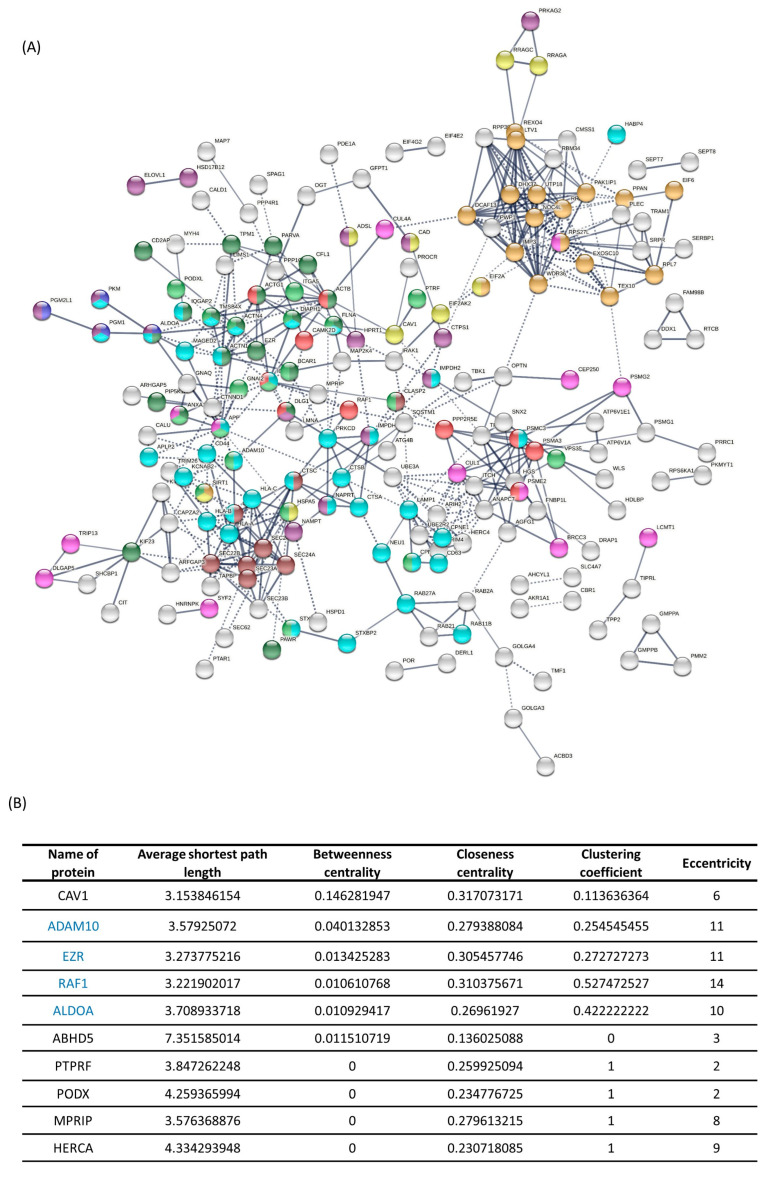

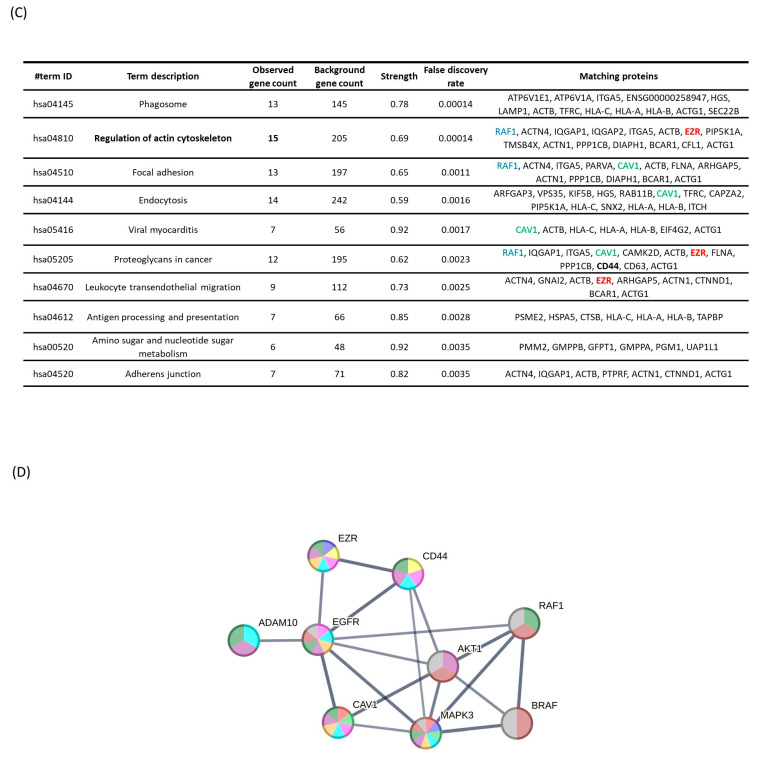
Bioinformatics analysis of combined proteomics datasets obtained by 2-DE/MALDI-TOF/TOF MS and LC–MS/MS to select new protein targets and associated cellular processes pertinent to the development of resistance to vemurafenib in BRAFV600E-mutated RKO colon cancer cells. (**A**) Network of interactions between the proteins differentially expressed in RKO colon cancer cells resistant to BRAFV600E targeting by vemurafenib (STRING). The PPI analysis included a combined list of proteins identified as overexpressed in the resistant cell line by 2-DE/MALDI-TOF/TOF MS and/or LC–MS/MS analysis. Nodes correspond to the dataset proteins and the edges to the combined evidence of the protein interactions (>0.7 evidence score). KMeans (10 clusters) clustering of the nodes was performed to refine visualisation of the connectivity patterns. The dashed lines depict edges connecting the clusters. Associations of the proteins with selected functional categories from GO Biological Functions ontologies are color-coded as follows: GO:0006903, vesicle targeting (*p* = 0.0018)—brown; GO:0042866, pyruvate biosynthetic process (*p* = 0.0459)—dark blue; GO:0042254, ribosome biogenesis (*p* = 2.39 × 10^−5^)—dark yellow; GO:0030036, actin cytoskeleton organisation (*p* = 1.50 × 10^−6^)—green; GO:0042594, response to starvation (*p* = 0.0083)—yellow; GO:0045055, regulated exocytosis (*p* = 1.39 × 10^−8^)—blue; GO:0009165, nucleotide biosynthetic process (*p* = 0.00059)—dark purple; GO:0040017, positive regulation of locomotion (7.12 × 10^−5^)—light green; GO:1901987, regulation of cell cycle phase transition (*p* = 0.0146)—purple; HSA-5673001 RAF/MAP kinase cascade (*p* = 0.0299)—red. (**B**) Cytoscape analysis. Topological connectivity characteristics of the selected proteins/potential targets. In bold—proteins with network connections and in the top 25 differential (MS) log_2_ fold change (*p* < 0.05); ADAM10 and RAF1—proteins with strong network connectivity and with low but positive differential (MS) log_2_ fold change; EZR and ALDOA—top differentially expressed proteins in MALDI data; average path length—average number of steps along the shortest paths for all possible pairs of network; betweenness centrality—the number of times a node acts as a bridge along the shortest path between two other nodes; closeness centrality—node’s average farness (inverse distance) to all other nodes; clustering coefficient—the degree to which nodes in a graph tend to cluster together; eccentricity—the degree of a node’s combined paths’ variation from being circular. (**C**) KEGG pathway enrichment analysis. (**D**) The network reconstructed in STRING shows the interconnection of the EZR (ezrin), CAV1, RAF1, ADAM10 and CD44 and the key functions involved in colorectal cancer. Network settings: 0.7 confidence, confidence view. Color-coded associations of the proteins with selected functional categories from GO Biological Functions, cellular components and KEGG pathways: Red—GO:0072584, caveolin-mediated endocytosis (0.00053); blue—GO:2000641, regulation of early endosome to late endosome transport (0.0029); purple—GO:0070161, anchoring junction (1.12 × 10^−5^); cyan—GO:0005925, focal adhesion (1.12 × 10^−5^); dark green—GO:0098805, whole membrane (0.00053); magenta—GO:0016323, basolateral plasma membrane(0.00091); orange—GO:0045121, membrane raft (0.0015); lime green—GO:0005901, caveola (0.0354); yellow—GO:0005902, microvillus (0.0399); maroon—hsa05218, melanoma (8.75 × 10^−9^); grey—hsa05210, colorectal cancer (8.75 × 10^−9^).

The PPI network (Figure 1A) for proteins with positive fold change ratio was subjected to clustering analysis to reveal potential functional modules and mapping by GO Biological Functions ontologies. As can be seen in Figure 1A, RAF/MAP kinase cascade-associated functions are central to the network linking the network modules of highly cross-connected nodes enriched in categories of ribosomal biogenesis (dark yellow), actin cytoskeletal organisation and positive regulation of locomotion (green), vesicle targeting (brown) and regulated exocytosis (light blue). Nucleotide biosynthetic processes (dark purple), regulation of cell phase transition (purple) and response to starvation are the least modular and are associated with different parts of the network. The largest meta-cluster of interconnected and cross-mapped functional nodes, central to the network, represented a strong association with cytoskeleton dynamics (locomotion, exocytosis, vesicle targeting). More detailed analysis of the network architecture aimed to select highly differentially expressed proteins with the strongest outreach that may be particularly relevant for the development of vemurafenib resistance and could thus represent novel pharmacological targets. Six of the top 25 proteins differentially expressed by MS analysis were found to be integrated in the network and thus were selected for further connectivity analysis, where two top-ranked proteins from MALDI analysis (EZR and ALDO) and two proteins with low differential expression but visually strong network connectivity (RAF1 and ADAM10) were added to this prioritised candidate list (Figure 1B).

Following the PPI network generation from all the differentially expressed proteins in the STRING database, we analysed the topology of the reconstructed PPI network using the Cytoscape analytical application to narrow down the selection of a target for subsequent experimental validation (Figure 1B). The assessment was based on characteristics of the node’s average path length (average number of steps along the shortest paths for all possible pairs of network nodes, which represents a measure of the efficiency of information or mass transport on a network), betweenness centrality (the number of times a node acts as a bridge along the shortest path between two other nodes), closeness centrality (node’s average farness (inverse distance) to all other nodes is a way of detecting nodes that are able to spread information very efficiently through a graph), clustering coefficient (the degree to which nodes in a graph tend to cluster together) and eccentricity (the degree of node’s combined paths’ variation from being circular—bigger eccentricities are less curved).

From this analysis (Figure 1B), Raf-1 proto-oncogene, serine/threonine kinase (RAF1), caveolin 1 (CAV1) and ezrin (EZR) emerged as the top ones in terms of average short path, meaning that information transports most efficiently through these nodes in the reconstructed network. This corresponds well to the top ranking of CAV1 and EZR in betweenness centrality and closeness centrality characteristics and of RAF1 and EZR in eccentricity. ADAM10 ranks second-top in betweenness centrality and eccentricity and is next to the top three in ranking by other characteristics. Overall, we can conclude that from the pre-selected nodes, CAV1, EZR, RAF1 and ADAM10 have the shortest distances to all other nodes and a central position in the highly connected part of the network, where CAV1 and EZR are also the top-ranked differentially expressed proteins detected by two different proteomics methods.

KEGG pathway enrichment analysis (Figure 1C) by means of the STRING analytical application also showed that the largest portion of identified proteins whose expression is higher in resistant cells (upregulated dataset) were significantly enriched in the regulation of the actin cytoskeleton with ezrin, amongst several other regulatory proteins such as CAV1 and RAF1, being placed in several relevant functional categories. The same analysis also revealed the relevance of CD44, classified under the proteoglycans in cancer functional terms, in association with vemurafenib resistance (Figure 1C). CD44, also being overexpressed in the resistant cells, is a cell-adhesion molecule known to interact with ezrin to form a complex that plays important roles in regulating tumour–endothelium interactions, cell migration, cell adhesion, tumour progression and metastasis [14].

A network reconstructed from the selected functions shows a strong integration with the functions involved in colon cancer and melanoma, namely AKT1, EGFR and MAPK3 (Figure 1D). Functional mapping of this network by the GO terms Biological Function and Cellular Component suggests a joint involvement of EZR and CAV1 in different stages of endocytosis and membrane trafficking and functional processes associated with the membrane rafts and basolateral membrane-associated anchoring junctions and focal adhesion sites. EZR and CD44 were also defined as the only functions in the network associated with microvilli (Figure 1D).

Altogether, bioinformatics analysis puts ezrin and caveolin-1 forward as highly ranked proteins significantly associated with vemurafenib resistance in colon cancer cells.

### 2.3. Increased Ezrin Expression Is Associated with Vemurafenib-Resistant Phenotype in BRAFV600E-Mutated RKO Colon Cancer Cells

**Figure 2:**
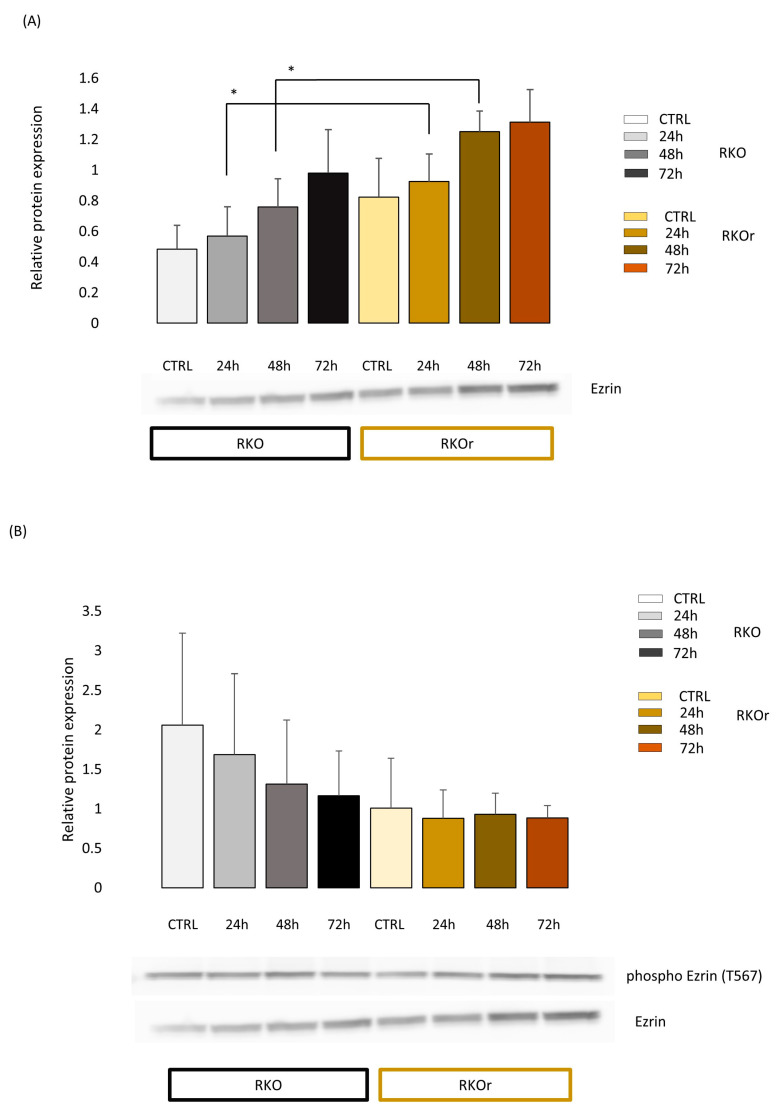
Western blot analysis of total ezrin (**A**) and phosphorylated ezrin (**B**) protein expression in vemurafenib-sensitive (parental, RKO) and -resistant (RKOr) colon cancer cells cultured in the absence (CTRL) and presence of vemurafenib (3 µM) for indicated time periods (24 h, 48 h, 72 h). The expression levels of ezrin and phospho-ezrin phosphorylated at threonine 567 were measured by densitometry analysis using the Quantity One 1-D Analysis Software version 4.6.6. The level of ezrin was normalised to the total amount of proteins loaded in the lane (total protein normalisation), whereas the level of phospho-ezrin was normalised to ezrin. The data represent the mean and standard deviation obtained from three independent biological experiments. Statistical significance (*p* < 0.05) is denoted with an asterisk.

Bioinformatics findings prompted us to further examine the involvement of ezrin and caveolin-1 in mediating vemurafenib resistance in RKO colon cancer cells. Ezrin is a member of the ezrin/radixin/moesin family of proteins and acts as a crosslinker between the plasma membrane and the actin cytoskeleton [15]. Ezrin is present in a closed inactive state in the cytoplasm and is activated by phosphorylation-induced conformational change where phosphorylation on threonine-567 (T567) is indispensable for binding to the F-actin cytoskeleton and enables linking of the actin cytoskeleton to the cell membrane [16]. We observed an increased total protein expression level of ezrin in resistant relative to sensitive RKO cells under baseline conditions (in the absence of vemurafenib) (Figure 2A) albeit without statistical significance. Importantly, exposure to vemurafenib at 3 µM (corresponding to the IC50 concentration of vemurafenib measured in sensitive cells) induced a significant rise in the total ezrin level in resistant cells relative to their sensitive counterparts after 24 and 48 h, and the same trend persisted after a 72 h treatment (Figure 2A). Interestingly, the level of phospho-ezrin (T567) was not significantly different between resistant and sensitive cells regardless of treatment conditions (Figure 2B). 

Similarly, longer culturing times (48 and 72 h) induced a significant upregulation of ezrin gene expression in the resistant cells under the baseline conditions, and the same expression pattern was sustained when cells were treated with vemurafenib (Supplementary Figure S1). 

To sum up, the upregulation of ezrin expression at the gene and protein level is suggested as an inherent feature of vemurafenib-resistant phenotype which could be additionally augmented by treatment with low-dose vemurafenib.

Caveolins are a family of membrane proteins involved in the formation of the plasma membrane invaginations termed caveolae, which play important roles in cellular trafficking and intracellular signaling, often via direct interaction with specific binding partners [17]. Caveolin-1, the best-characterised isoform of this family in terms of its roles in cancer, can function either as tumour suppressor or tumour promoter depending on the cancer type, cellular context and conditions [17]. We detected a markedly increased protein expression level of caveolin-1 in vemurafenib-resistant in comparison with sensitive RKO cells grown without vemurafenib (Supplementary Figure S2), which corroborates our proteomics findings showing upregulation of caveolin-1 in resistant cells. Strikingly, exposure to vemurafenib dramatically increased caveolin-1 expression levels in sensitive cells peaking at 72 h, while vemurafenib induced a rise in caveolin-1 expression in resistant cells that was much smaller in scale. Given its dual roles in cancer that impart both anti- and pro-apoptotic functions to caveolin-1, this protein was excluded as a putative target for counteracting vemurafenib resistance from further in vitro validation studies.

### 2.4. Pharmacological Inhibition of Ezrin by NSC305787 Increases the Response of the Resistant RKO Colon Cancer Cells to Vemurafenib and Concurs with a Downregulation of CD44 Expression and Inhibition of AKT/c-Myc Signaling

**Figure 3:**
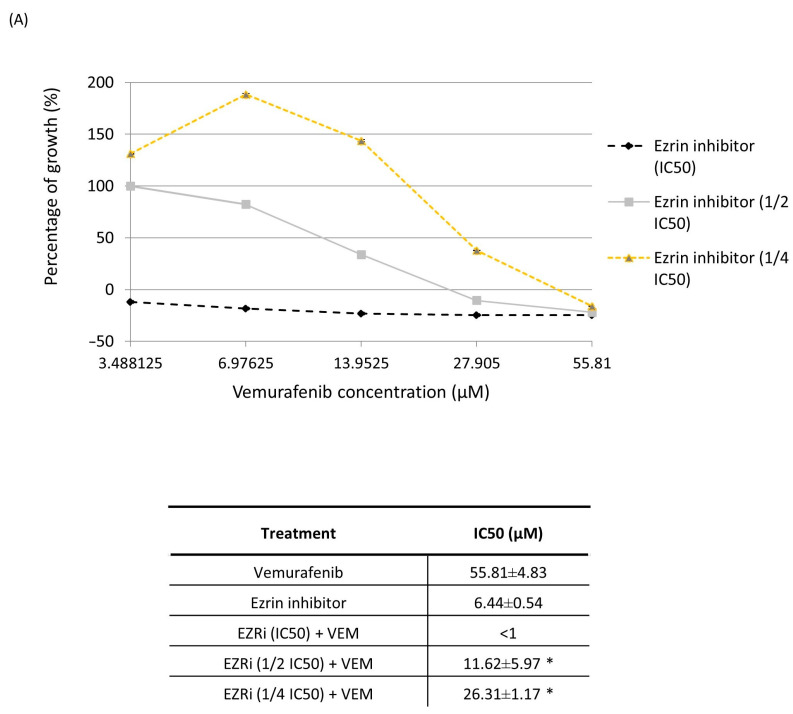

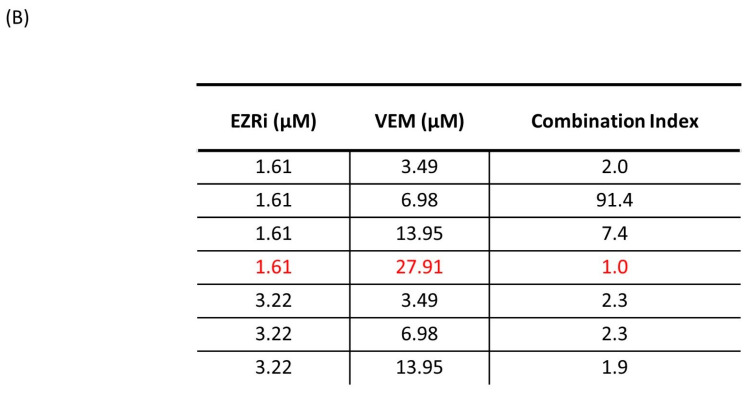
Anti-proliferative effect of combination treatment with vemurafenib and ezrin inhibitor NSC305787 in vemurafenib-resistant RKO colon cancer cells after a 72 h treatment period. (**A**) Concentration-dependent effect of combination treatment on cell viability measured by MTT assay. IC50 values were calculated using linear regression analysis and the results were statistically analysed by ANOVA with Tukey’s post hoc test, and statistically significant differences (*p* < 0.05) are indicated by an asterisk. Data were obtained from three independent biological experiments. (**B**) The combination index (CI) was calculated for each drug combination from growth inhibition curves using CompuSyn software (version 1.0), where CI = 1, <1 and >1 indicate additive effect, synergism and antagonism, respectively. Data were obtained from three independent biological experiments. The treatment combination exerting an additive anti-proliferative effect is highlighted in red and was selected for further functional studies.

Since ezrin has emerged as a promising target suggested by bioinformatics analyses, we further sought to investigate the possibility of restoring the sensitivity of the resistant RKO cells to vemurafenib by targeting ezrin. To test this hypothesis, we treated the resistant cells with five different concentrations of vemurafenib (starting from IC50 concentration down to four different sub-toxic concentrations) in combination with three concentrations of pharmacological ezrin inhibitor NSC305787 at IC50 and two sub-toxic concentrations and measured the anti-proliferative effect of treatment combinations by MTT assay. Selective ezrin inhibitor NSC305787 is an experimental anti-cancer agent that has been so far evaluated only in pre-clinical studies which showed that NSC305787 has acceptable toxicity and pharmacokinetics profiles in murine models [12]. Our results revealed that pharmacological inhibition of ezrin markedly increased the cell-growth-inhibitory activity of vemurafenib in the resistant RKO cells in a concentration-dependent manner when compared to a single-agent treatment with vemurafenib (Figure 3A). Importantly, ezrin inhibitor was able to restore the sensitivity of the resistant cells to vemurafenib even at low sub-toxic concentrations.

To determine the nature of the response for each tested drug combination in the resistant RKO cells, we calculated the combination index (CI) values using CompuSyn software, where CI < 1, =1 and >1 indicated synergism, additive effect and antagonism, respectively. Surprisingly, CI for the most tested treatment combinations was above 1, which indicates drug antagonism. However, one combination treatment with sub-toxic concentrations of ezrin inhibitor at 1.61 µM (1/4 IC50) and vemurafenib at 27.91 µM (1/2 IC50) produced an additive anti-proliferative effect in the resistant RKO cells with CI = 1.0 (Figure 3B). This combination treatment was thus selected for all subsequent mechanistic studies in the resistant RKO colon cancer cells.

**Figure 4:**
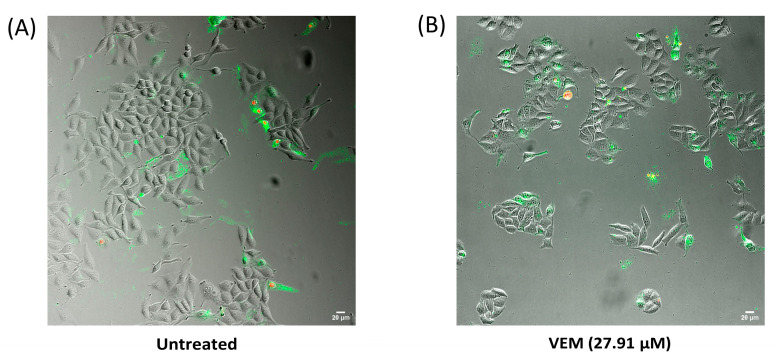

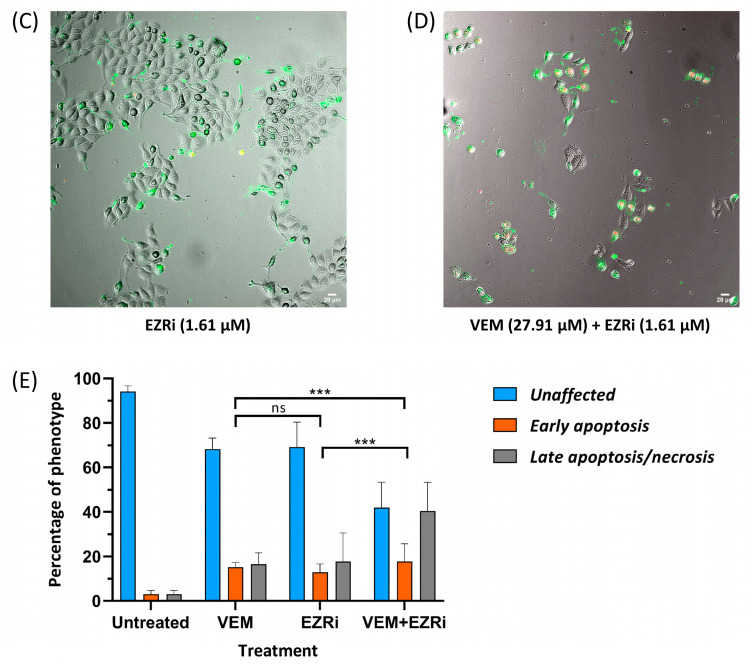
Pro-apoptotic effect of combination treatment with vemurafenib and ezrin inhibitor NSC305787 in vemurafenib-resistant RKO colon cancer cell line after a 48 h treatment period. (**A**–**D**) Representative images of Annexin V assay performed to detect induction of apoptosis when cells were treated with either single agents or their combination at indicated concentrations for 48 h. Confocal microscopy images were taken at 20× magnification: (**A**) untreated cells; (**B**) cells treated with vemurafenib (VEM); (**C**) cells treated with EZR inhibitor (EZRi); (**D**) cells treated with dual therapy (VEM + EZRi); (**E**) quantification of the percentage of unaffected cells (Ann−/PI−), early apoptotic cells (Ann+/PI−) and late apoptotic/primary necrotic cells (Ann+/PI+). Data are representative of two independent biological experiments performed in two replicates. Ann—Annexin V; PI—propidium iodide. Values represent the mean ± SD. The number of visual fields was 8 for all the treatments at both replicates. Two-way ANOVA with Tukey’s post hoc test was used for statistical analysis where only the most significant differences between the groups were marked by three asterisks (*p* ≤ 0.001). ns = non-significant.

We then investigated the effect of co-treatment with vemurafenib and the ezrin inhibitor on induction of apoptosis at a combination of their concentrations producing additive effect, as described above. As can be seen from Figure 4, there was a pronounced reduction in the percentage of unaffected cells concomitant with a marked increase in the percentage of late apoptotic/necrotic cells in the combination treatment in comparison with single-agent vemurafenib or ezrin inhibitor, which demonstrates that ezrin inhibition increases the anti-survival effect of vemurafenib in the resistant RKO cells.

**Figure 5:**
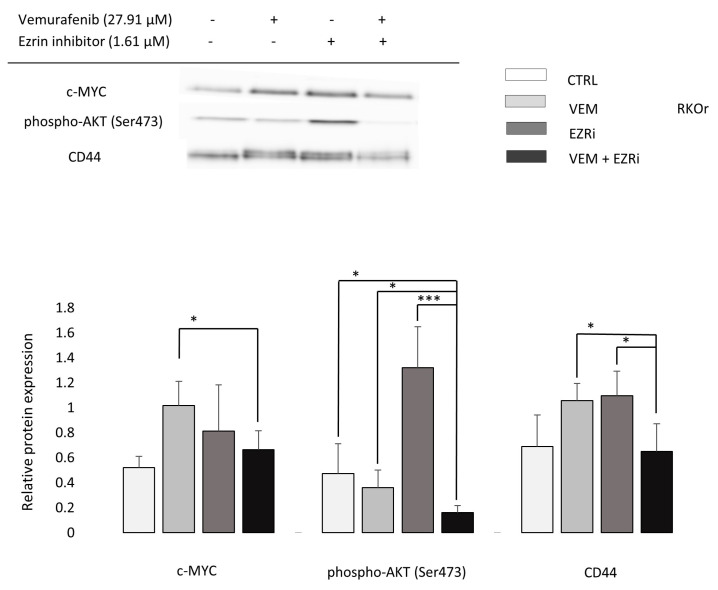
Western blot analysis of protein targets potentially associated with the chemosensibilisation effect of combination treatment with vemurafenib and ezrin inhibitor NSC305787 in vemurafenib-resistant RKO colon cancer cells after a 72 h treatment period. Relative protein expression levels of selected proteins were measured by densitometry analysis using Quantity One software and normalised to the total amount of protein loaded in the lane (total protein normalisation). The data represent the mean and standard deviation obtained from three independent biological experiments. Statistical significance (*p* < 0.05) is denoted with an asterisk, and *p* ≤ 0.001 with three asterisks. CTRL—untreated/control cells; VEM—vemurafenib/27.91 µM; EZRi—ezrin inhibitor/1.61 µM; VEM + EZRi—27.91 µM vemurafenib + 1.61 µM ezrin inhibitor.

Finding that the combination of vemurafenib and the ezrin inhibitor at selected sub-toxic concentrations improves the anti-proliferative and pro-apoptotic activity of vemurafenib in the resistant RKO cells has spurred further study to investigate the potential mechanism behind the observed chemosensibilisation effect. The PPI network analysis in STRING (Figure 1D) indicated the interconnection of ezrin with CD44, which is congruent with literature data reporting that ezrin binds to cell-adhesion molecule CD44 implicated in cancer-cell migration and metastasis [14]. Another interesting protein candidate emerging from the ezrin network appeared to be AKT, the component of the PI3K/AKT signaling pathway activated by ezrin in different cancer types [12] and previously demonstrated to mediate de novo vemurafenib resistance in BRAFV600E-expressing RKO cells [18]. Finally, c-Myc expression was evaluated for combined anti-proliferative effects based on the previous findings showing that ezrin positively regulates c-Myc protein levels in prostate cancer cells by activating the AKT signaling pathway downstream from ezrin that controls c-Myc expression at the gene and protein level [19]. Our data clearly showed that the combination treatment with vemurafenib and ezrin inhibitor significantly downregulated protein expression levels of CD44 in comparison with both single-agent treatments (Figure 5). Similarly, the combination treatment induced a significant reduction in the levels of phospho-AKT (Ser473) indicative of abrogation of AKT activity when compared to both individual treatments and to untreated cells. A similar regulation pattern of protein expression was observed for c-Myc whose level was significantly decreased in the combination treatment relative to individual treatments (Figure 5). To sum up, the increased anti-cancer effect of vemurafenib in the resistant RKO cells induced by ezrin inhibitor was accompanied by a downregulation of CD44 expression and an inhibition of AKT/c-Myc signaling.

### 2.5. Increased Ezrin Expression Accompanies the Development of Resistance to Vemurafenib in A375 Melanoma Cells Carrying the BRAFV600E Mutation

**Figure 6:**
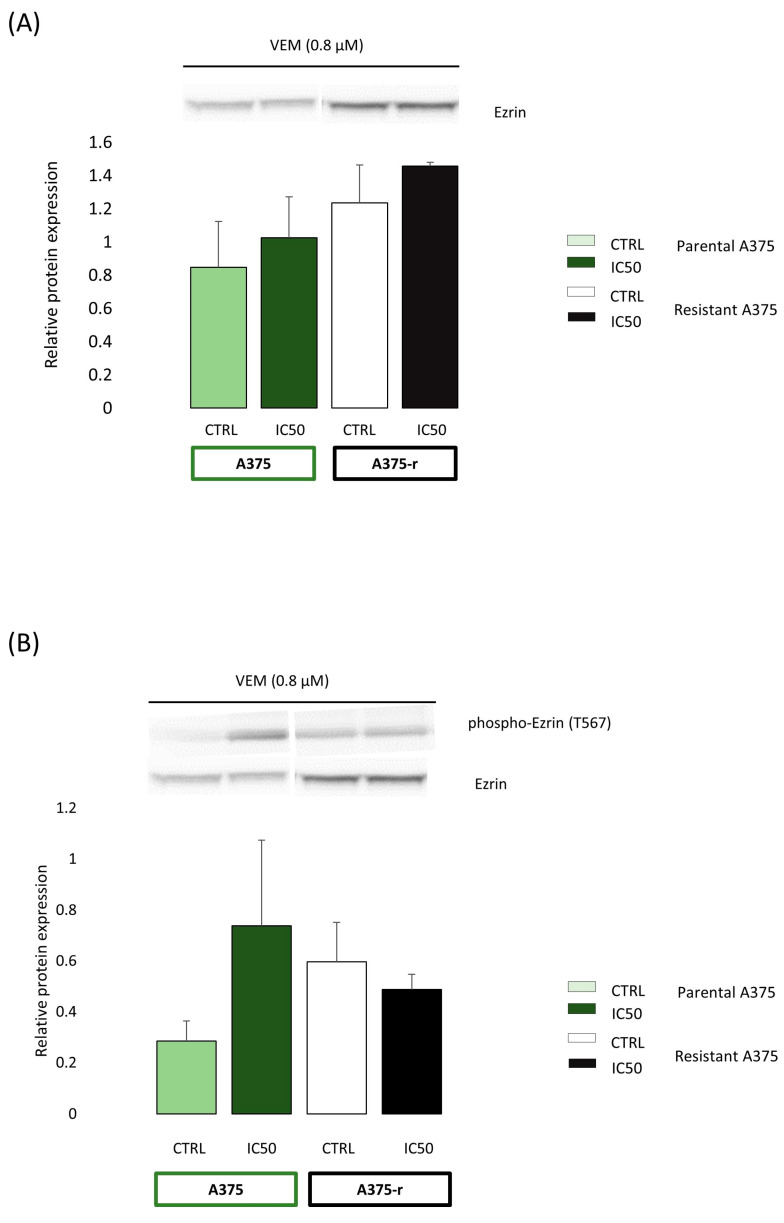
Western blot analysis of total ezrin (**A**) and phosphorylated ezrin (**B**) protein expression in vemurafenib-sensitive (parental, A375) and vemurafenib-resistant (A375-r) melanoma cells carrying the BRAFV600E mutation in the absence (CTRL) and in the presence of 0.8 µM vemurafenib for 72 h. The expression levels of ezrin and phospho-ezrin phosphorylated at threonine 567 were measured by densitometry analysis using the Quantity One software. The level of ezrin was normalised to the total amount of proteins loaded in the lane (total protein normalisation), whereas the level of phospho-ezrin was normalised to ezrin. The data represent the mean and standard deviation obtained from three independent biological experiments.

In vitro observation of the involvement of ezrin in vemurafenib resistance in BRAFV600E-mutated RKO colon cancer cells prompted us to examine whether ezrin plays an important role in acquired resistance to BRAFV600E inhibition in another BRAFV600E-mutated cancer such as melanoma. Previous literature data already provide an evidence of the role of the actin cytoskeleton in mediating adaptive resistance to vemurafenib in BRAFV600E-mutant melanoma cells [20,21]. Knowing that ezrin is involved in the regulation of the actin cytoskeleton organisation, we analysed the expression levels of total ezrin and phospho-ezrin (T567) in vemurafenib-sensitive and vemurafenib-resistant A375 melanoma cells harbouring the BRAFV600E mutation under the baseline culture conditions and after exposure to 0.8 µM vemurafenib (corresponding to the IC50 concentration of vemurafenib measured in sensitive melanoma cells) for 72 h (Figure 6). We observed a marked trend towards increased expression of total ezrin and phospho-ezrin in resistant melanoma cells under baseline conditions, which indicates that increased abundance of ezrin could be potentially linked with the vemurafenib-resistant phenotype in BRAFV600E-mutated melanoma cells. 

### 2.6. Pharmacological Inhibition of Ezrin by NSC305787 Potentiates Anti-Proliferative and Pro-Apoptotic Effects of Vemurafenib in the Resistant A375 Melanoma Cells in a Synergistic Manner

**Figure 7:**
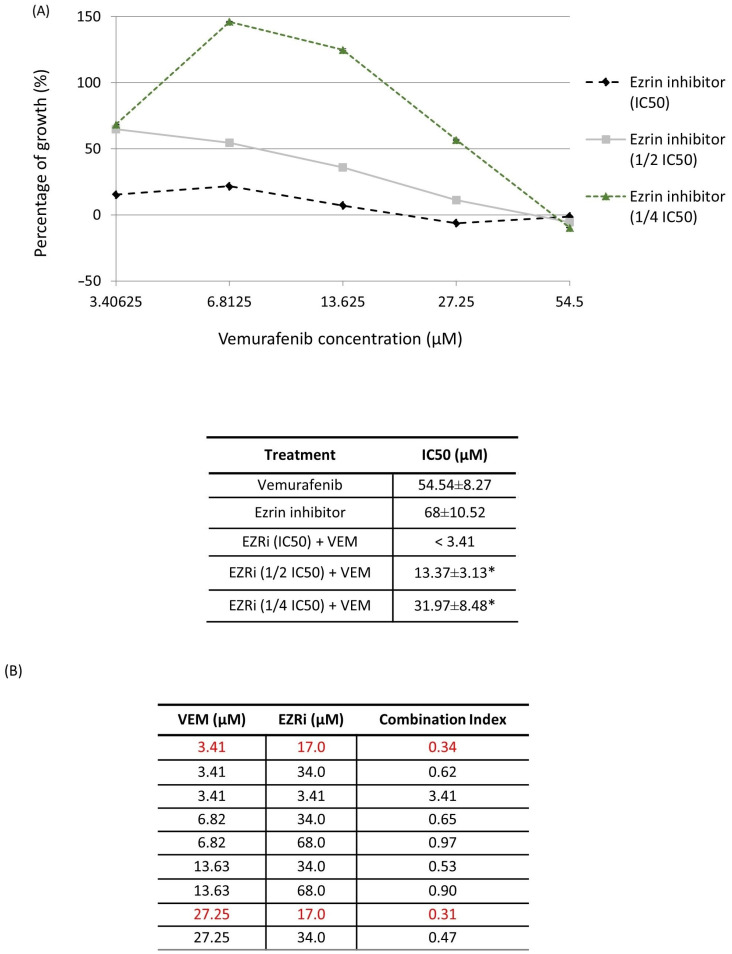
Anti-proliferative effect of the combination treatment with vemurafenib and ezrin inhibitor NSC305787 in vemurafenib-resistant A375 melanoma cells carrying the BRAFV600E mutation after a 72 h treatment period. (**A**) Concentration-dependent effect of combination treatment on cell viability measured by MTT assay. IC50 values were calculated using linear regression analysis and the results were statistically analysed by ANOVA with Tukey’s post hoc test, and statistically significant differences (*p* < 0.05) are indicated by an asterisk. Data were obtained from three independent biological experiments. (**B**) The combination index (CI) was calculated for each drug combination from growth inhibition curves using CompuSyn software, where CI = 1, <1 and >1 indicate additive effect, synergism and antagonism, respectively. Data were obtained from three independent biological experiments. Combinations of concentrations exerting the most potent synergistic effect are highlighted in red.

**Figure 8:**
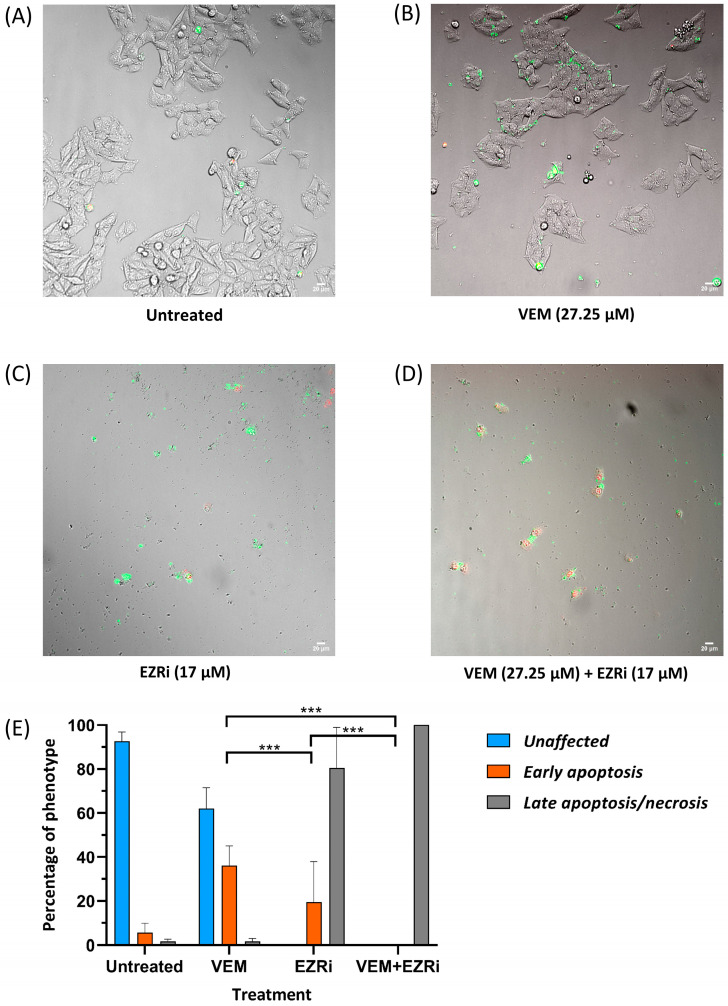
Pro-apoptotic effect of the combination treatment with vemurafenib and ezrin inhibitor NSC305787 in vemurafenib-resistant A375 melanoma cell line after a 48 h treatment period. (**A**–**D**) Representative images of Annexin V assay performed to detect induction of apoptosis when cells were treated with either single agents or their combination at indicated concentrations for 48 h. Confocal microscopy images were taken at 20x magnification: (**A**) untreated cells; (**B**) cells treated with vemurafenib (VEM); (**C**) cells treated with EZR inhibitor (EZRi); (**D**) cells treated with dual therapy (VEM + EZRi); (**E**) quantification of the percentage of unaffected cells (Ann−/PI−), early apoptotic cells (Ann+/PI−) and late apoptotic/primary necrotic cells (Ann+/PI+). Data are representative of two independent biological experiments performed in two replicates. Ann—Annexin V; PI—propidium iodide. Values represent the mean ± SD. The number of visual fields was 8 for all the treatments at both replicates. Two-way ANOVA with Tukey’s post hoc test was used for statistical analysis where only the most significant differences between the groups were marked by three asterisks (*p* ≤ 0.001).

We next evaluated the ability of ezrin inhibitor NSC305787 to increase anti-proliferative and anti-survival effects of vemurafenib in A375 melanoma cells with acquired resistance to vemurafenib. Expectedly, ezrin inhibitor potentiated the anti-proliferative effect of vemurafenib even in the low sub-toxic concentration range (Figure 7A). Furthermore, we tested nine combinations of toxic and sub-toxic concentrations of vemurafenib and NSC305787 for their ability to reduce cell viability of resistant melanoma cells. Surprisingly, we found that all tested concentration combinations of the two agents exerted a synergistic growth-inhibitory effect on vemurafenib-resistant melanoma cells in a range of their sub-toxic concentrations (Figure 7A,B). Among these, the most potent synergistic anti-proliferative effect was elicited by two sub-toxic concentrations of vemurafenib (3.41 µM and 27.25 µM corresponding to 1/16 IC50 and 1/2 IC50, respectively), in combination with one sub-toxic concentration of the ezrin inhibitor (17 µM corresponding to 1/4 IC50) (Figure 7B). The combination of 27.25 µM vemurafenib and 17 µM NSC305787, having the lowest combination index, was further evaluated for its ability to augment vemurafenib-induced apoptosis in resistant melanoma cells (Figure 8). Results from Annexin V assay revealed that treatment of the resistant A375 cells with a combination of vemurafenib and ezrin inhibitor completely depleted the unaffected-cell population in comparison with vemurafenib. Importantly, combination treatment induced a marked upsurge in the late apoptotic/necrotic cell population relative to treatment with single agents, in particular with vemurafenib (Figure 8). Altogether, these findings show that ezrin inhibition potentiates the pro-apoptotic effect of vemurafenib in the resistant A375 cells.

## 3. Discussion

In the present paper, we present data from proteomics profiling of BRAFV600E-mutated colon cancer cells with acquired resistance to the BRAFV600E inhibitor vemurafenib. Here, we integrated datasets of differentially expressed proteins identified by two complementary proteomics technologies including 2-DE coupled with MALDI-TOF MS and label-free quantitative LC–MS/MS, which were then subjected to bioinformatics analyses for biological data interpretation and identification of the key candidate proteins and pathways related to vemurafenib resistance. Obtained results revealed the actin cytoskeleton organisation as one of the most significant upregulated features of the vemurafenib-resistant phenotype and pointed to ezrin, the cross-linker between plasma membrane proteins and the actin cytoskeleton, as the most relevant putative protein target for pharmacological intervention. We specifically confirmed a time-dependent increase in ezrin expression at the gene and protein levels in vemurafenib-resistant in comparison with vemurafenib-sensitive RKO colon cancer cells harbouring the BRAFV600E mutation under baseline conditions and after exposure to low concentration of vemurafenib. Importantly, pharmacological inhibition of ezrin by the small molecule inhibitor NSC305787 potentiated anti-proliferative and pro-apoptotic effects of vemurafenib in the resistant cells in an additive manner, which was accompanied by a downregulation of CD44 expression and suppression of the pro-survival AKT/c-Myc signaling.

Previous studies similarly reported the role of upregulated ezrin expression in the mechanisms underlying the resistance to chemotherapy in different cancer types including breast cancer [22,23,24], tongue squamous cell carcinoma [25] and osteosarcoma [26], and demonstrated that ezrin suppression by genetic or pharmacological manipulation could increase chemotherapeutic sensitivity. Although the high expression level of ezrin was previously correlated with colorectal cancer invasion, metastasis and worse prognosis indicating the role of ezrin in the pathogenesis of colorectal cancer [27,28], no data have been presented so far on its role in modulating the response and development of resistance to chemotherapy drugs in colon cancer. Interestingly, high ezrin expression was previously correlated with BRAFV600E mutation status in colorectal cancer since high ezrin immunohistochemical staining was found predominantly in BRAFV600E-mutated tumours (52%) in comparison with BRAF wild-type tumours (17%) [29]. Our findings thus indicating the role of ezrin as a novel target for chemosensitisation in BRAFV600E-mutated colon cancer may be particularly relevant in light of recent clinical evidence demonstrating short-term efficacy of clinically approved combination therapy with EGFR/BRAF inhibitors in BRAFV600E-mutant metastatic colorectal cancer patients due to rapid development of resistance [30], which necessitates the development of novel therapeutic strategies for BRAFV600E-mutated colon cancer. In this respect, ezrin inhibitors might be further investigated within pre-clinical and clinical set-ups as an adjunct to BRAF inhibitors in treating BRAFV600E-mutant colon cancer.

The BRAFV600E mutation has been shown to affect the tumour immune microenvironment in colorectal cancer [31], melanoma [32] and glioblastoma [33], and intrinsic resistance to BRAFV600E inhibition may stem from the changes in the tumour microenvironment resulting in the activation of the compensatory signals and/or reactivation of the targeted pathway [33].

The observation that the actin-cytoskeleton linker protein ezrin was overexpressed in the resistant colon cancer cells motivated us to examine its possible involvement in mediating vemurafenib resistance in melanoma cells since the role of actin-cytoskeleton organisation and remodeling was previously demonstrated in transition to vemurafenib-resistant phenotype in BRAFV600E mutant melanoma in vitro [20,34]. We have specifically found increased levels of ezrin in vemurafenib-resistant melanoma cells carrying the BRAFV600E mutation under baseline conditions and in the presence of low concentration of vemurafenib (0.8 µM). Importantly, the combination of ezrin inhibitor NSC305787 and vemurafenib at low sub-toxic concentrations exerted synergistic growth-inhibitory and apoptosis-inducing effects in resistant cells, which indicates the role of ezrin in regulating vemurafenib resistance in BRAFV600E mutant melanoma.

Collectively, our study has revealed that ezrin is commonly involved in vemurafenib resistance in BRAFV600E-mutated colon cancer and melanoma cells. It was interesting to note that the combination treatment with the ezrin inhibitor and vemurafenib induced different modes of cytostatic effects by exerting synergistic effect in melanoma vs. additive effect in colon cancer cells. This discrepancy could be, at least partially, ascribed to different genetic backgrounds and diverse inherent sensitivity of these two cancer cell types to tested drugs, which resulted in the selection of a different concentration range of the drugs evaluated in the two cell lines. Nevertheless, BRAFV600E-mutated colon cancer and melanoma cells share some molecular similarities accompanying the switch to a drug resistant phenotype that could result from the common BRAFV600E genotype as well as from a BRAFV600E-directed transcriptional regulatory pathway that mediates epigenetic silencing commonly observed in colorectal cancer and melanoma [35].

## 4. Materials and Methods

### 4.1. Cell Culturing Conditions

Human colon carcinoma cell line RKO and human melanoma cell line A375 both harbouring the BRAFV600E mutation were purchased from the American Type Culture Collection (Manassas, VA, USA). Both RKO and vemurafenib-resistant RKO cells were maintained in Eagle’s Minimum Essential Medium (Capricorn Scientific, Ebsdorfergrund, Germany) supplemented with 10% fetal bovine serum (Capricorn Scientific, Germany), 2 mM L-glutamine (Capricorn Scientific, Germany), penicillin (100 U/mL) (Capricorn Scientific, Germany) and streptomycin (100 µg/mL) (Capricorn Scientific, Germany) in humified atmosphere with 5% CO_2_ at 37 °C. Melanoma cell line A375 and its vemurafenib-resistant counterpart were maintained in Dulbecco’s Modified Eagle Medium (Capricorn Scientific, Germany) supplemented with 10% fetal bovine serum (Capricorn Scientific, Germany), 2 mM L-glutamine (Capricorn Scientific, Germany), penicillin (100 U/mL) (Capricorn Scientific, Germany), streptomycin (100 µg/mL) (Capricorn Scientific, Germany) and 1 mM sodium pyruvate (Capricorn Scientific, Germany) in humified atmosphere with 5% CO_2_ at 37 °C.

### 4.2. Two-Dimensional Gel Electrophoresis (2-DE) and Image Analysis

Parental and vemurafenib-resistant RKO cells were seeded in 100 mm Petri dishes at a density of 1 × 10^6^ cells and cultured for 48 h. Cells were then lysed using a lysis buffer containing 7M urea, 2M thiourea, 4% (*w/v*) CHAPS, 10 (*w/v*) DTT (Sigma-Aldrich, St. Louis, MO, USA) supplemented with protease-inhibitor cocktails (Roche, Basel, Switzerland). A total of 700 µg proteins was dissolved in 330 µL 2-DE rehydration buffer containing 7M urea, 2M thiourea, 4% (*w/v*) CHAPS, 10% (*w/v*) DTT and 0.2% (*w/v*) BioLyte^®^ 3/10 Ampholyte (BIO-RAD, Hercules, CA, USA). Samples were loaded onto 17 cm, pH 3–10NL ReadyStrip™ IPG Strips (BIO-RAD, USA) and the strips were overlaid with mineral oil (BIO-RAD, USA). Isoelectric focusing was performed using PROTEAN IEF cell (BIO-RAD, USA) under the following conditions: 50 V for 14 h (active rehydration), 250 V gradual for 30 min, 500 V gradual for 30 min, 1000 V gradual for 30 min, 10,000 V gradual for 3 h, and 10,000 V rapid for 43,000 VHours. In the second dimension, proteins were resolved by 12% SDS-polyacrylamide gels PROTEAN II XL cell (BIO-RAD, USA). The gels were stained by Coomassie Brilliant Blue G-250 (Sigma-Aldrich, USA) overnight, followed by washing in milliQ water. Gel images were taken by ChemiDoc XRS+ Imager (BIO-RAD, USA), and image analysis was conducted using Progenesis SameSpots 4.0 (TotalLab, Newcastle upon Tyne, UK). The experiment was performed in six individual biological replicates for each cell type. ANOVA analyses followed by Tukey’s post hoc test were carried out to identify statistically significant differences in protein abundance between the datasets.

### 4.3. MALDI-TOF/TOF Mass Spectrometry Analysis

Protein spots of interest were excised from the gel, cut into approximately 1 mm^3^ cubes, and destained using acetonitrile (Honeywell, Charlotte, NC, USA) and 100 mM ammonium bicarbonate (ABC, Sigma-Aldrich, St. Louis, MO, USA). Samples were then dried in a vacuum concentrator to complete dryness, followed by in-gel tryptic digestion performed by incubating the gel in 50 mM ABC containing 10 ng/µL trypsin (sequencing grade, Promega, Madison, WI, USA) at 4 °C overnight. The following day, supernatant was collected, and tryptic peptides were extracted in three steps consisting of incubation in 65% acetonitrile/5% formic acid solution, MiliQ water and 100% acetonitrile with shaking and sonication. In between steps supernatants were collected and subsequently dried in a vacuum concentrator, redissolved in 0.1% trifluoroacetic acid and purified by C18 ZipTip (MerckMillipore, Burlington, MA, USA) according to the manufacturer’s instructions. Each sample was then mixed with matrix solution containing α-cyano-4-hydroxycinnamic acid (0.3 g/L CHCA in the solution containing 2:1 ethanol: acetone, *v/v*) at the ratio of 1:10. A total amount of 1 µL of the mixture containing sample/matrix solution was spotted onto the MALDI plate (AnchorChip 800 μm, Bruker Daltonics, Bremen, Germany) and kept at room temperature until crystallisation was completed. An UltrafleXtreme MALDI-TOF/TOF mass spectrometer (Bruker Daltonics, Billerica, MA, USA) was used to perform MS analyses in the reflector mode in the *m/z* range of 700–3500 Da. The MS spectra were externally calibrated using a mixture of Peptide Calibration Standard and Protein Calibration Standard I (Bruker Daltonics, USA) at the ratio of 1:5. FlexControl 3.4 software (Bruker Daltonics, USA) was applied to acquire and process spectra. FlexAnalysis 3.4 (Bruker Daltonics, USA) was applied to perform protein database searches. Proteins were identified using the Mascot 2.4.1 search engine (Matrix Science, London, UK). The search parameters were as follows: Enzyme: trypsin; Fixed modifications: Carbamidomethylation on cysteine; Variable modifications: Oxidation on methionine; Protein mass: Unrestricted; Peptide mass tolerance: ±50 ppm; Maximum missed cleavage: 2.

### 4.4. LC–MS/MS

#### 4.4.1. LC–MS/MS Sample Preparation

Cells were lysed in buffer containing 4% sodium dodecyl sulfate (SDS) before boiling at 95 °C for 10 min. Twenty µg of proteins was taken and reduced with 5 mM dithio-threitol for 30 min at room temperature followed by alkylation with 15 mM iodoacetamide at 50 °C for 30 min in the dark. Samples were processed using the single-pot solid-phase enhanced sample preparation (SP3) [36,37]. Protein purification, digest and peptide clean-up were performed using the KingFisher Flex System (Thermo Fisher Scientific, Waltham, MA, USA) and Carboxylate-Modified Magnetic Particles (GE Life Sciences, Piscataway, NJ, USA; GE65152105050250, GE45152105050250), as described previously [38]. After the digest, peptides were re-solubilised in 15 µL of MS sample buffer (3% acetonitrile, 0.1% formic acid).

#### 4.4.2. LC–MS/MS Data Acquisition

Mass spectrometry analysis was performed on a Q Exactive HF mass spectrometer (Thermo Scientific) equipped with Digital PicoView source (New Objective, Littleton, MA, USA) and coupled with M-Class UPLC (Waters, Milford, CA, USA). For each sample, 2 μL of peptides was loaded on a commercial ACQUITY UPLC M-Class Symmetry C18 Trap Column (100 Å, 5 µm, 180 µm × 20 mm, Waters) followed by ACQUITY UPLC M-Class HSS T3 Column (100 Å, 1.8 µm, 75 µm × 250 mm, Waters). The peptides were eluted at a flow rate of 300 nL/min with a gradient from 5 to 24% B in 80 min and 24 to 36% B in an additional 10 min. The column was cleaned after the run by increasing to 95% B and holding 95% B for 10 min prior to re-establishing loading conditions. Samples were measured in randomised order. The mass spectrometer was operated in data-dependent mode (DDA) on the top-12 most abundant ions using Xcalibur (tune version 2.9), with spray voltage set to 2.3 kV, funnel RF level at 60% and heated capillary temperature at 275 °C. Full-scan MS spectra (350−1500 *m/z*) were acquired at a resolution of 120,000 at 200 *m/z* after accumulation to an automated gain control (AGC) target value of 100,000 or for a maximum injection time of 50 ms. Precursors with an intensity above 4500 were selected for MS/MS and subjected to a dynamic exclusion of 30 s. Ions were isolated using a quadrupole mass filter with 1.2 *m/z* isolation window and fragmented by higher-energy collisional dissociation (HCD) using a normalised collision energy of 28%. MS2 spectra were recorded at a resolution of 35,000 and a maximum injection time of 54 ms. Charge-state screening was enabled, and singly, unassigned charge states and charge states higher than seven were excluded. The mass spectrometry proteomics data were handled using the local laboratory information management system (LIMS) [39].

#### 4.4.3. LC–MS/MS Data Analysis

The acquired raw MS data were processed by MaxQuant (version 1.6.2.3), followed by protein identification using the integrated Andromeda search engine [40]. Spectra were searched against the Uniprot Homo sapiens reference proteome (taxonomy 9606, canonical version from 9 July 2019), concatenated to its reversed decoyed fasta database and common protein contaminants. Carbamidomethylation of cysteine was set as fixed modification, while methionine oxidation and N-terminal protein acetylation were set as variable. Enzyme specificity was set to trypsin/P allowing a minimal peptide length of 7 amino acids and a maximum of 2 missed cleavages. MaxQuant Orbitrap default search settings were used. The maximum false discovery rate (FDR) was set to 0.01 for peptides and 0.05 for proteins. Label-free quantification was enabled and a 2 min window for match-between-runs was applied. In the MaxQuant experimental design template, each file is kept separate in the experimental design to obtain individual quantitative values. Protein fold changes were computed based on Intensity values reported in the proteinGroups.txt file. A set of functions implemented in the R package SRMService [41] was used to filter for proteins with 2 or more peptides and to normalise the data with a modified robust z-score transformation and to compute *p*-values using the *t*-test with pooled variance. If all measurements of a protein were missing in one of the conditions, a pseudo fold change was computed replacing the missing group average by the mean of 10% smallest protein intensities in that condition.

The mass spectrometry proteomics data have been deposited to the ProteomeXchange Consortium via the PRIDE (http://www.ebi.ac.uk/pride (accessed on 25 May 2023)) partner repository with the dataset identifier PXD042499. 

### 4.5. Bioinformatics Analysis of Proteomics Datasets

#### 4.5.1. Networks Reconstruction and Analysis by STRING

The STRING database (STRING Consortium) [42] comprises SIB—Swiss Institute of Bioinformatics, CPR—Novo Nordisk Foundation Center Protein Research and EMBL—European Molecular Biology Laboratory. It integrates and statistically validates weights of protein–protein interactions from multiple reliable sources and includes 24,584,628 proteins and 3,123,056,667 total protein interactions (with 530,027,879 interactions at a medium confidence or better (score ≥ 0.400)). In STRING, each protein–protein interaction is annotated with one or more ‘scores’. Importantly, these scores do not indicate the strength or the specificity of the interaction. Instead, they are indicators of confidence, i.e., how likely STRING judges an interaction to be true, given the available evidence. All scores rank from 0 to 1, with 1 being the highest possible confidence. A score of 0.5 would indicate that roughly every second interaction might be erroneous (i.e., a false positive).

In STRING evidence view each edge represents scored evidence on a protein-specific and meaningful pairwise functional contribution to a shared function (not necessarily a physical interaction). Interactions can be known (experimentally determined, retrieved form curated databases) or predicted from genome neighborhood, gene fusion or gene co-occurrence, co-expression, co-mentioning in literature or protein homology, and are indicated by an edge color. In a confidence view one weighted edge represents the combined evidence scores. In our analysis we used refined data setting a high STRING interaction score filter of 0.7. IDs of all the proteins differentially expressed in the resistant vs. sensitive cells (log_2_ (fold change ratio) and *p*-values < 0.05) from LC–MS and MALDI proteomics datasets were used for the network generation based on all protein/gene interaction evidence available from the STRING database (experimental, co-expression, database-co-mentioning, etc.), and with a selected confidence threshold >0.7. All disconnected nodes were disregarded. Tables listed all the connected nodes and the combined scores of all the edges were imported to Cytoscape 3.8.2.

In reconstructed and analysed networks nodes represent all the proteins produced by a single, protein-coding gene locus (splice isoforms or post-translational modifications are being collapsed). Location of the nodes in a network corresponds to ‘centrality’ index of a node, so that nodes with a strongest impact on a network are presented in the network’s core—its central part.

As a part of the STRING’s functional analysis application, selected functional categories were projected onto a network via color-coding of the nodes listed in a selected ontology category of GO Biological Process, KEGG pathways or Reactome. To define network modules with the strongest inter-connectivity, K-means clustering of the nodes was performed by an application of STRING-integrated clustering tool. STRING network functional enrichment analysis was used to determine functional groups in KEGG and Go-‘Biological processes’ terms, specific for each network.

#### 4.5.2. Network Reconstruction and Analysis by Means of Cytoscape Platform

Cytoscape 3.8.2 [43] was used to determine topological characteristics of connectivity of the proteins of interest in a reconstructed network. Networks were created by means of STRING using all the protein/gene interaction evidence available from STRING database (experimental, co-expression, databases-co-mentioning, etc.), and with a selected confidence threshold > 0.7. All disconnected nodes were disregarded. The tables listed all the connected nodes and the combined scores of all the edges were imported to Cytoscape 3.8.2. IDs of all the proteins differentially expressed in the subject samples vs. controls (positive and negative log_2_ (fold change ratio) and *p*-values < 0.05 from MS and MALDI proteomics) were used for these network generation.

The Analyze Network application was executed to retrieve the topological characteristics of the network in respect to each node. Edge-weighted spring-embedded layout was applied to demonstrate the STRING-calculated connectivity aspects of the network. Prefuse force-directed OpenCL Layout (with a selected Edge Betweennes option) were used to enforce demonstration of a node connectivity.

Topological analysis of the network implied analysis of the following characteristics: Average path length is defined as the average number of steps along the shortest paths for all possible pairs of network nodes. It is a measure of the efficiency of information or mass transport on a network. Betweenness centrality quantifies the number of times a node acts as a bridge along the shortest path between two other nodes. Closeness centrality is a way of detecting nodes that are able to spread information very efficiently through a graph. The closeness centrality of a node measures its average farness (inverse distance) to all other nodes. Nodes with a high closeness score have the shortest distances to all other nodes. A Clustering coefficient is a measure of the degree to which nodes in a graph tend to cluster together. Eccentricity shows how much a path varies from being circular. Bigger eccentricities are less curved.

The number of nodes used in this analysis was relatively small; however, the connectivity exceeded the expected based on an average/random connectivity of all the proteins recorded in STRING database and had a connectivity reliability of 10–16 with about 50% of connections exceeding the expected number in each reconstructed net. This score was not been affected by a presence or absence of the resistance-relevant protein set added to the extended networks, which suggests a strong functional interconnection of the majority of the differentially expressed proteins detected by the proteomics analysis. The networks generated in this analysis do not include all the proteins potentially expressed in the tissue (as it may have been done with a data from a quantitative transcriptomics or a full proteomics profile for each sample set), which makes more detailed and precise modelling of the functional outcomes of an inhibition of a particular selected node not feasible.

### 4.6. Cell Viability Assay

Cell viability was assessed using MTT assay. Briefly, cells were seeded onto 96-well microtiter plates at a density of 3000 cells/well. The subsequent day, cells were treated with selected test agents in five 10-fold serial dilutions (10^−4^–10^−8^ µM) and incubated for 72 h. MTT assay was performed according to the manufacturer’s instructions (Sigma-Aldrich, St. Louis, MO, USA). After the 72 h treatment period, cells were incubated with MTT reagent for 3 h in the dark followed by the addition of dimethyl sulfoxide (Sigma-Aldrich, St. Louis, MO, USA). Absorbance was measured using a Tecan SPARK multimode microplate reader (Tecan Life Sciences, Männedorf, Switzerland) at 570 nm. Inhibitory and lethal concentrations (IC50 and LC50, respectively), were calculated using linear regression analysis.

Combined treatments of resistant RKO cell line were performed using five 2-fold serial dilutions of vemurafenib IC50 concentrations (55.81, 27.91, 13.96, 6.98 and 3.49 µM) in combination with three 2-fold dilutions of IC50 concentrations of ezrin inhibitor (NSC305787) (6.44, 3.22 and 1.61 µM), and the combined treatments of resistant A375 cell line were performed using five 2-fold serial dilutions of vemurafenib IC50 concentrations (54.50, 27.25, 13.63, 6.81 and 3.41 µM) in combination with three 2-fold dilutions of IC50 concentrations of ezrin inhibitor (68, 64 and 17 µM). All inhibitors are commercially available and were purchased from MedChemExpress (MedChemExpress, Monmouth Junction, NJ, USA). After treatment for 72 h, cell viability was assessed using MTT assay, as described above. Combination index (CI) was calculated using CompuSyn software [44]. Combination index (CI) values that correspond to <1, =1 and >1 indicate on synergy, additivity and antagonism, respectively. Each experiment was performed as a tetraplicate in three biological experiments.

### 4.7. Western Blot Analysis

For Western blot analysis, cells were seeded in 6-well plates at a density of 1.5 × 10^5^ cells per well and cultured for the indicated period in the presence, absence or combination of vemurafenib and ezrin inhibitor. After incubation cells were lysed using RIPA buffer (25 mM Tris-HCl (pH 7.4), 1% NP-40, 0.5% sodium deoxycholate, 0.1% SDS, 150 mM NaCl) supplemented with phosphatase and protease inhibitor cocktails (Roche, Switzerland). A total of 50 µg proteins was resolved on 12% SDS polyacrylamide gels and transferred onto PVDF membranes (Bio-Rad, USA). Membranes were blocked in 5% bovine serum albumin (PAN-Biotech, Aidenbach, Germany) prepared in TBST buffer, and probed with primary antibodies against ezrin, caveolin, CD44 and c-MYC (all diluted at 1:1000, Cell Signaling Technologies, Danvers, MA, USA), and phospho-ezrin T567 (dilution 1:1000; St. John’s Laboratory, London, UK) overnight at 4 °C. The following day, membranes were washed with TBST buffer and probed with secondary antibody goat anti-rabbit (at dilution 1:2000, Cell Signaling Technologies). Protein bands were visualised using Amersham™ ECL™ Prime Western Blotting Detection Reagent (Cytiva, Little Chalfont, UK) and Amersham Imager 600 (GE Healthcare, Chicago, IL, USA). Relative protein expression was analysed by Quantity One 1-D Analysis Software version 4.6.6 (Bio-Rad, Hercules, CA, USA). Briefly, membranes were stained with Coomassie and visualised using Amersham Imager 600 prior to incubation with an antibody. Images were then densitometrically analysed to obtain the total protein load per lane, and membranes were washed with TBST, blocked, and probed with antibodies, as described above. Densitometry data of protein bands was normalised against total protein load per lane. Statistical analysis was performed by the two-tailed *t*-test (*p* < 0.05).

### 4.8. Quantitative Real-Time PCR

Total cellular RNA from two independent experiments was extracted (RNeasy Mini Kit, Qiagen, Hilden, Germany) and quality of extracted RNA verified by electrophoresis (RNA ScreenTape Analysis, Agilent TapeStation 4200, Agilent Technologies, Santa Clara, CA, USA). After quality-control concentration of extracted RNA was determined (Qubit RNA BR Assay Kit, Thermo Fisher Scientific, Waltham, MA, USA), 1 µg of RNA was treated with DNase I (DNase I, Thermo Scientific, USA) according to the manufacturer’s instructions and 2 µL of DNase-treated RNA (200 ng) was reverse transcribed (FIREScript^®^ RT cDNA synthesis KIT, Solis Bodyne, Tartu, Estonia). For the expression analysis by quantitative real-time PCR (qPCR) cDNA was diluted in H_2_O (ratio 1:1) and 1 µL was used in the reaction with Sybr Green reagent (Brilliant III Ultra-Fast SYBR Green QPCR Master Mix with Low ROX, Agilent, USA) on an Agilent AriaMx Real-Time PCR System 5 (Agilent, USA). All the reactions were performed in triplicate, and expression of EZR gene was normalised to the housekeeping gene GAPDH using the ∆∆Ct method.

List of primers used (Macrogen, Maastricht, The Netherlands):
PrimerSequence (5′-3′)Ta/°CFor_EZRTGTGGTACTTTGGCCTCCAC60Rev_EZRTCTCCTTCCTGACCTCCTGG60For_GAPDHTCAAGGCTGAGAACGGGAAG60Rev_GAPDHCGCCCCACTTGATTTTGGAG60

### 4.9. Apoptosis Detection

Vemurafenib-resistant RKO and vemurafenib-resistant A375 cells were seeded at 6000 cells per well and 24 h post seeding treated with corresponding drugs. Vemurafenib-resistant RKO cells were treated with vemurafenib (27.91 µM), ezrin inhibitor (1.61 µM), and their combination, whereas vemurafenib-resistant A375 cells were treated with vemurafenib (27.25 µM), ezrin inhibitor (17 µM) and their combination. Forty-eight hours post treatment, cells were washed and stained with the Annexin V-FITC Apoptosis Staining/Detection Kit (ab14085, Abcam, Cambridge, UK) as per the manufacturer’s instructions. Images were taken with the ImageXpress Micro Confocal High-Content Imaging System (Molecular Devices, San Jose, CA, USA) using Nikon 20x Ph1 S Plan Fluor ELWD objective (Nikon) under 50 µm slit IXConfocal module disk geometry.

Images of the same visual field were obtained under transmitted light (TL50), FITC and Cy3 filters, different channels merged in ImageJ version 1.53t (National Institutes of Health, Bethesda, MD, USA) and cells counted and divided into categories (unaffected cells, early apoptosis, late apoptosis/necrosis).

## 5. Conclusions

Based on the results presented herein, we propose that the inhibition of ezrin could be further tested and explored as a novel strategy to counteract resistance to the BRAFV600E inhibitors in tumours harbouring the BRAFV600E mutation. Indeed, we have shown that ezrin overexpression is associated with vemurafenib-resistant phenotype in BRAFV600E-mutated colon cancer and melanoma cells and that pharmacological inhibition of ezrin restores the sensitivity of resistant cells to vemurafenib in vitro. Accordingly, pre-clinical and clinical studies designed to explore the benefits of combined BRAF inhibitors and drugs targeting the ezrin-regulated actin cytoskeleton as a novel therapeutic approach for BRAFV600E-mutated cancers should be encouraged.

## Data Availability

The data presented in this study are openly available in ProteomeXchange Consortium via the PRIDE (http://www.ebi.ac.uk/pride (accessed on the 25 May 2023)) partner repository with the dataset identifier PXD042499.

## Supplementary Materials

The supporting information can be downloaded at: https://www.mdpi.com/article/10.3390/ijms241612906/s1.
