# Supplementary material for: Ezrin Inhibition Overcomes Acquired Resistance to Vemurafenib in BRAFV600E-Mutated Colon Cancer and Melanoma Cells In Vitro
Source: Int J Mol Sci. 2023 Aug 17;24(16):12906. doi: 10.3390/ijms241612906 (PMC10454476; doi:10.3390/ijms241612906)
Supplement: Supplementary file 1 [file ijms-24-12906-s001.zip › Supplementary Figures S1 and S2.pdf]

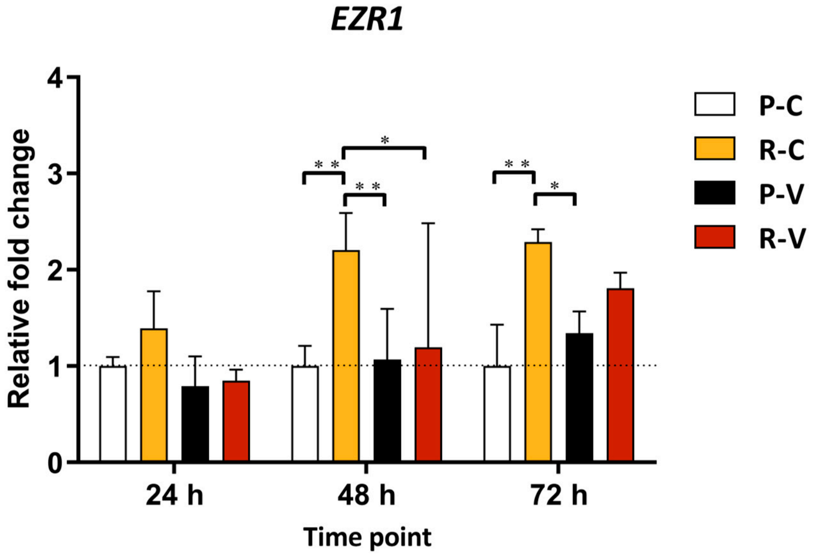

Figure S1. Ezrin gene expression analysis by real-time quantitative PCR in vemurafenib-sensitive and resistant RKO cells in the absence and in the presence of vemurafenib (3 µM) for indicated time periods. Data represent mean and standard deviations from two independent biological experiments carried out in technical duplicates, where statistical significance is denoted with an asterisk. P (parental RKO cells), R (vemurafenib-resistant RKO cells), C – untreated cells (control), V- treatment with vemurafenib. Statistical significance ($p < 0.05$) is denoted with an asterisk, ($p < 0.01$) is denoted with two asterisks.

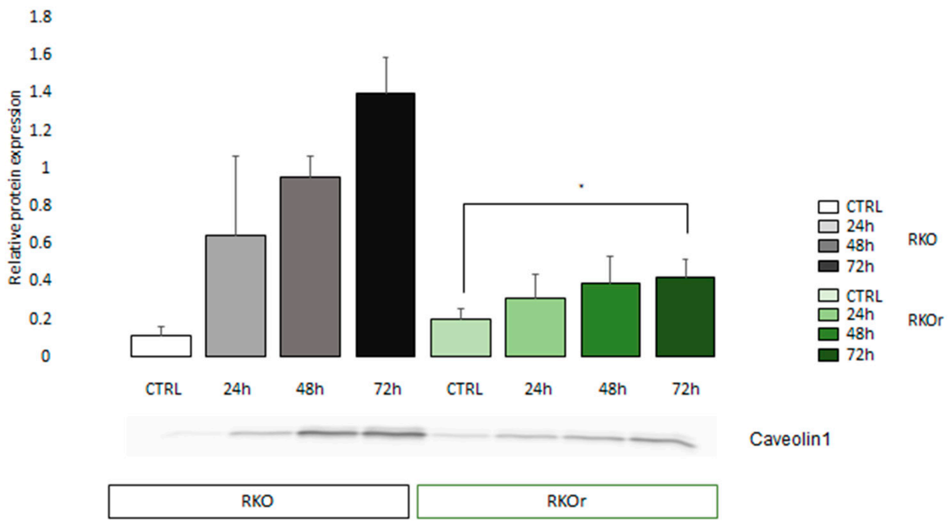

Figure S2. Western blot analysis of caveolin-1 expression in vemurafenib-sensitive (parental) and resistant RKO colon cancer cells in the absence and in the presence of vemurafenib (3 µM) for indicated time periods. Relative protein expression was measured by densitometry analysis using Quantity One software. Data represent mean and standard deviation obtained from three independent biological experiments. Statistical significance ($p < 0.05$) is denoted with an asterisk. CTRL indicates untreated (control) cells.
